# Conservative Management of Unicystic Ameloblastoma of Mandible Evolving from Dentigerous Cyst in a Paediatric Patient: A Case Report

**DOI:** 10.30476/dentjods.2022.94689.1804

**Published:** 2023-06-01

**Authors:** Verma Meenal, Verma Nikhil, Astekar Madhusudan

**Affiliations:** 1 Dept. Oral and Maxillofacial Pathology, Geetanjali Dental and Research Institute, Udaipur (313001) Rajasthan, India; 2 Dept. Prosthodontics and Crown and Bridge, Principal, Geetanjali, Dental and Research Institute, Udaipur (313001) Rajasthan, India; 3 Dept. Oral Pathology and Microbiology, Institute of Dental Sciences, Bareily (243006) Uttar Pradesh, India

**Keywords:** Ameloblastoma, Unicystic ameloblastoma, Dentigerous cyst, Mural Ameloblastoma, Vicker and Gorlin’s criteria

## Abstract

Massive cystic lesions involving a mandible always present a diagnostic and treatment challenge. Unicystic ameloblastoma (UA) is a variant of ameloblastoma encompassing about 6% of ameloblastomas. They represent cystic lesions that reveal clinical and radiographic features of a cyst, but the histopathological features demonstrate a typical ameloblastomatous epithelium lining the cyst. It is a variant of ameloblastoma, usually having clinical and radiographic similarities with dentigerous cysts, hence posing preoperative diagnostic difficulties. Adult treatment protocol cannot be applied to the pediatric population since resection may cause an alteration in craniofacial development leading to functional and esthetical damage, which can directly affect their quality of life. A more conservative approach of enucleating the lesion seems to be a promising treatment modality of UA in the pediatric age group. We present a case of mural variant of UA arising from dentigerous cyst in an 8-year-old male patient.

## Introduction

Ameloblastoma is the most common locally aggressive benign tumor affecting the mandible. It has many histological subtypes out of which unicystic ameloblastoma (UA) is comparatively less encountered variant possessing slow growth but locally destructive potential [ 1 - 2 ]. UA refers to those cystic lesions, which show clinicoradiographic facade of odontogenic cysts and tumors, but histologically present a typical ameloblastomatous lining epithelium with/without luminal and or mural proliferations thus challenging the surgeons in terms of diagnosis and treatment especially in young children. Incisional biopsy specimens may not always be representative of the true nature of UA, which may have a negative bearing on the treatment plan [ 3 ]. Usually, UA respond well to conservative management, so aggressive protocols like segmental resection should be avoided in children [ 4 ]. Here we report a case of mural UA arising from dentigerous cyst lining in an eight-year-old male patient, which was managed through conservative enucleation.

## Case Presentation

**Figure 1:**
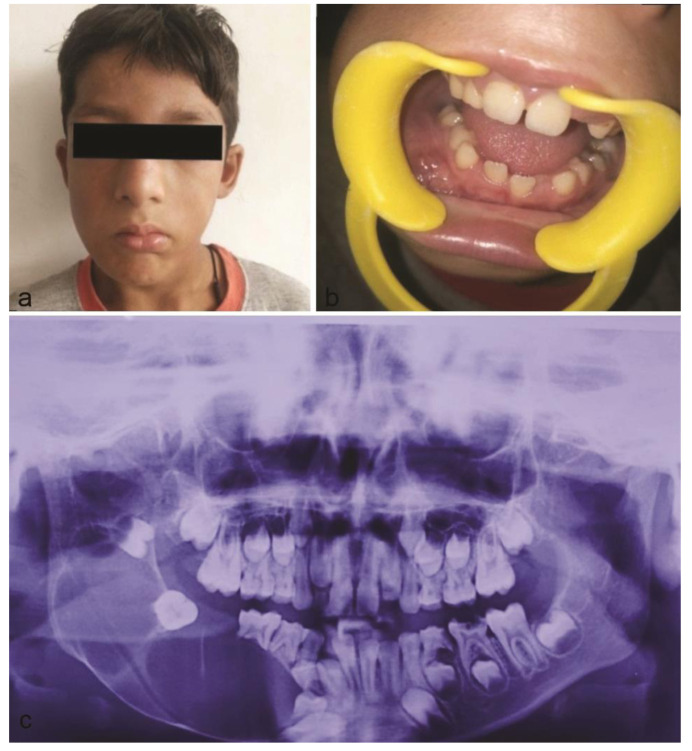
**a:** Extra oral image of patient showing diffuse swelling on right side of the face, **b:** Intraorally obliterating swelling present in right buccal vestibule, **c:** A panoramic radiograph showing well-defined multilocular radiolucency with respect to right mandibular posterior region associated with knife-edge pattern of root resorption in 85 and 46

An 8-year-old male patient, reported to our institute with the chief complaint of pain and swelling on right back side of the face since 10 -12 days following a fall from a tree. History revealed that the patient was apparently asymptomatic 6 months back with gradual onset of swelling on right side of face. Extra oral examination revealed a diffuse hard and indurated swelling on right side of the face, extending anteriorly from the corner of the mouth to the ramus molar region posteriorly and superiorly extending from the infraorbital region, to inferior border of the mandible. There was associated difficulty in opening the mouth and facial asymmetry. Intraoral examination revealed a mixed dentition, carious primary molars in first, second and third quadrants and a non-tender swelling on lower right posterior region, measuring between 5cm×2cm, obliterating buccal vestibule. Panoramic radiograph, revealed a large well-defined unilocular radiolucency in relation to markedly displaced, developing, unerupted 45 associated with knife-edge pattern of root resorption in 85 and 46 (Figure 1). Provisional diagnosis of dentigerous cyst and differential diagnosis of odontogenic keratocyst and ameloblastoma was given. Complete hemogram showed all normal parameters.

**Figure 2:**
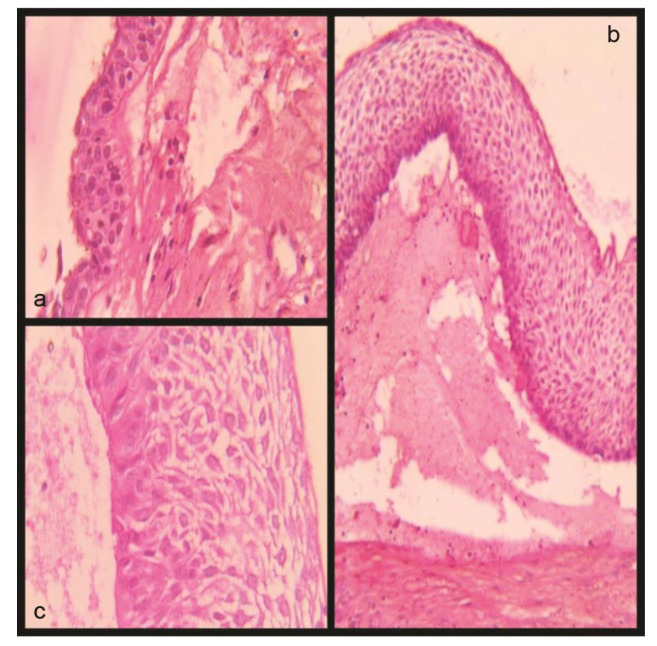
**a:** Cystic lumen lined by stratified squamous non-keratinized epithelium, **b** and **c:** Neoplastic ameloblastomatous transformation of epithelium with basal palisading, nuclear hyperchromatism, reverse polarity, spongiosis in superficial layer consistent with stellate reticulum like cells. (Hematoxylin& Eosin stain, original magnification 10× (B), 40 (C))

**Figure 3:**
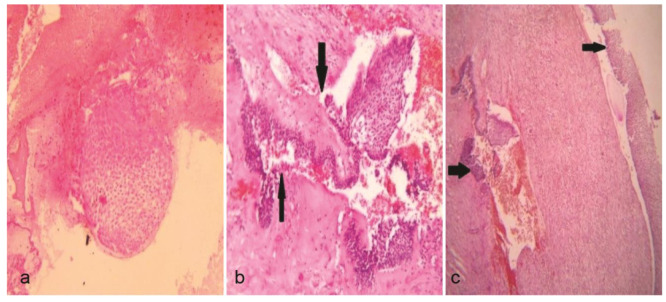
**a:** Ameloblastomatous epithelium projecting as intraluminal mass with plexiform pattern. (Hematoxylin and Eosin stain, original magnification 10×), **b:** Intramural islands of ameloblastomatous follicles with corresponding subfollicular hyalinization. (Black arrowhead) (Hematoxylin and Eosin stain, original magnification 40×), **c:** Luminal and mural components with the mural follicles showing cystic degeneration (Black arrowhead) (Hematoxylin and Eosin stain, original magnification 10×)

**Figure 4:**
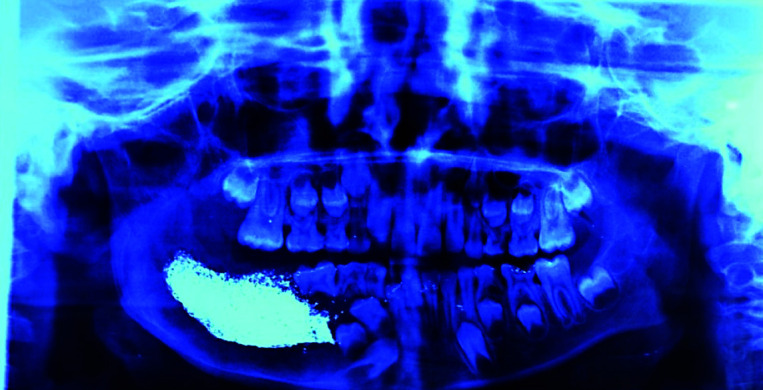
Post- operative panoramic radiograph with iodoform gauge

Surgical enucleation (with removal of two underdeveloped permanent molars and one premolar) by intraoral vestibular incision approach, followed by peripheral osteotomy preserving the inferior border of mandible, under general anesthesia was performed. Defect cavity was packed with iodoform glycerin-soaked gauze for proper granulation (Figure 2). Excisional biopsy specimens were processed. Microscopic examination of hematoxylin & eosin (H&E) stained sections revealed cystic lumen lined partly by thin non keratinized stratified squamous epithelium (suggestive of dentigerous cyst lining) transforming into focal areas of columnar basal cells showing hyperchromatic and palisading nucleus with reversal of polarity and superficial cells showing changes like presence of moderate to abundant pale, acidophilic vacuolated cytoplasm and round to oval vesicular nucleus corresponding with stellate reticulum, consistent with Vickers and Gorlin criterion for ameloblastomatous epithelium (Figure 3). In few areas lining epithelium was seen proliferating and projecting in form of intraluminal masses demonstrating an edematous, plexiform pattern. Underlying mural fibro cellular tissue revealed multiple neoplastic odontogenic epithelial islands, forming strands and follicles with ameloblastomatous changes, cystic degeneration, and corresponding hyalinization (Figure 4). Focal areas of thin lamellar bony trabeculae, patchy dystrophic calcifications, hemorrhage, and low to moderate amount of lympho-plasmacytic infiltrate were also noted. Correlating the histopathological findings with clinical and radiographic presentation, a final diagnosis of mural UA (UCA subgroup 1.2.3) arising from dentigerous cystic lining was established.

**Figure 5:**
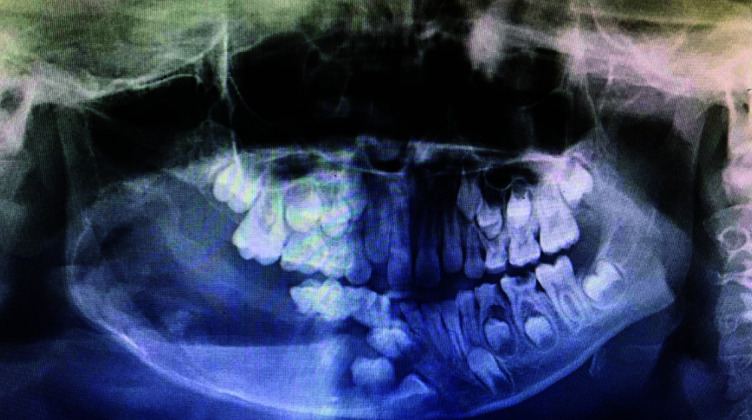
Post-operative panoramic radiograph at 8 months of follow up

**Figure 6:**
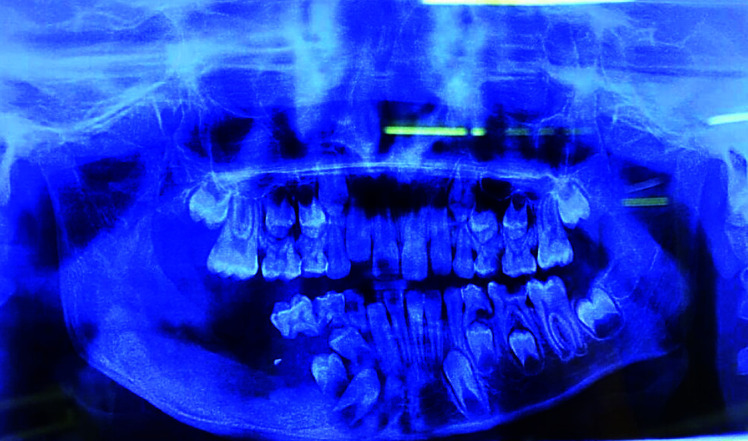
Post-operative panoramic radiograph at 11 months of follows up

**Figure 7:**
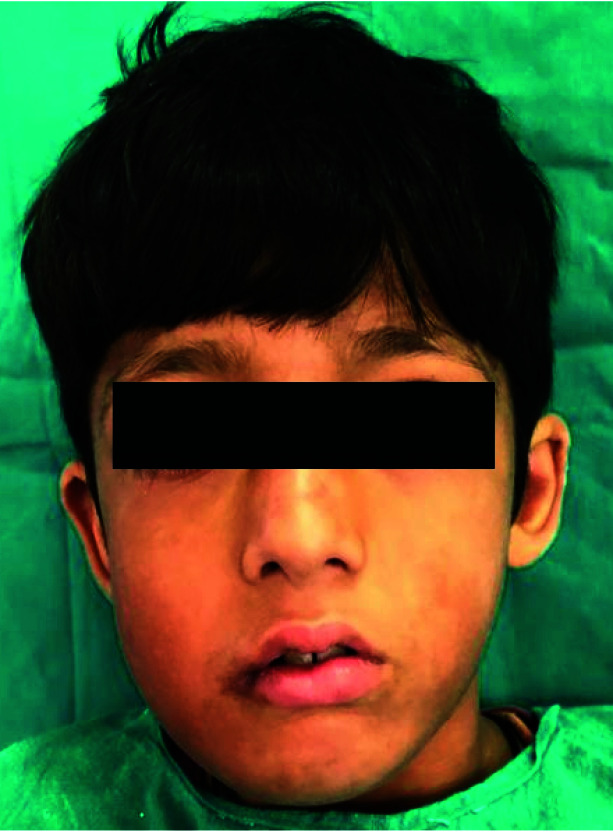
Facial profile after surgery

Patient was weekly followed up for initial 18 months and thereon at every 3 months; any possible recurrence was monitored by taking regular panoramic radiographs. Each time, surgical defect was irrigated using Betadine followed by glycerin soaked iodoform dressing for six months. During follow up visits the panoramic radiographs revealed a gradually reduction in defect cavity size and subsequent new bone filling the defect (Figures 5-6). After three years of follow up, patient was free of any complaints, with reduced defect cavity size, and new bone formation evident radiographically; normal healing was noted without any signs of recurrence till date (Figure 7).

## Discussion

UA is a variant of ameloblastoma, first described by Robinson and Martinez [ 1 ] considering the macro- and microscopic appearance, the lesion being essentially a well-defined, large monocystic cavity having a lining, focally composed of odontogenic ameloblastomatous epithelium. They may be associated with an unerupted tooth (dentigerous variant) or lack such association (nondentigerous variant) [ 1 ]. Their evolution from epithelial remnants of developing tooth, dentigerous cyst lining or from cystic degeneration of a solid ameloblas toma has long been debated. However, Ackermann *et al*. [ 2 , 5 ] and Robinson and Martinez [ 2 , 5 ] favored their origin from dentigerous cyst lining owing to common lineage through reduced enamel epithelium and its high neoplastic transformation probability secondary to irritants like extraction, trauma, and infections [ 2 , 5 ]. This pathophysiology corresponds with the current case. Dentigerous UAs have predilection for males (1.6: 1) and younger age (mean 16.5 years) in mandibular posterior ramus molar region while the nondentigerous UAs affect females (1.8: 1) generally in older age (mean 35.2 years) [ 2 ]. Present case matches with these specifics. Radiographically, UAs have two patterns including unilocular and multilocular, former being commonly associated with dentigerous variant of UAs [ 2 ].

Based on configuration and extent of ameloblastomatous component within the luminal cyst wall, four histologic subtypes of UAs are identified namely luminal UA (subgroup 1), luminal and intraluminal UA (subgroup 1.2), luminal intraluminal and intramural UA (subgroup 1.2.3), and luminal and intramural UA (subgroup 1.3). Amongst these, subgroups 1.2.3 and 1.3 call for a more aggressive treatment approach like a radical resection owing to their high recurrence rates [ 1 ]. Multiple histopathological sections study in our case guided towards the final diagnosis of UA with mural proliferation (subgroup 1.2.3).

The treatment of UAs is still being considered very controversial due to the incongruous use of the term “mural proliferation” [ 6 ], which is erroneously interpreted as increased recurrence rate, hence favoring radical resection [ 1 , 7 ]. In fact, the term “mural” describes the extent of ameloblastomatous epithelium penetrating connective tissue wall of a cyst and not that mural ameloblastoma has penetrated the epithelial lining of a cyst [ 6 , 8 ]. Mural UA is believed to recur following enucleation due to remnants of tumor cells in fibrous capsule [ 9 ]. Ameloblastoma developing in and limited to the cystic lining and ameloblastoma having micro invasions into the connective tissue wall of the cyst should be managed by enucleation whereas ameloblastoma with complete invasion of the connective tissue thickness should be treated with resection [ 6 , 10 ]. In the current case, conservative enucleation was treatment of choice with satisfactory and uneventful follow up of three years.

## Conclusion

This case report highlights the role of histopathologic examination and serial section study from multiple sites of representative specimen as the most sensitive tool for precise diagnosis of UAs and also advocates the conservative treatment approach as the choice of treatment in young children combined with close long term follow up with satisfactory results.

## Acknowledgements

Authors would like to thank Dr. Harvey Thomas (Prof.& Head, Dept. of OMFS, GDRI), Dr. Poorwa Sharma (Sr. Lecturer, Dept. of OMFS, GDRI)) for their referral and help in enabling the authors to follow up this case.

## Conflict of Interest

Authors declare that there are no conflicts of interest in this study.

## References

[1] Garcia NG, Oliveira DT, Rodrigues MT ( 2016). Unicystic ameloblastoma with mural proliferation managed by conservative treatment. Case Rep Pathol.

[2] Nagalaxmi V, Sangmesh M, Maloth KN, Kodangal S, Chappidi V, Goyal S ( 2013). Unicystic mural ameloblastoma: an unusual case report. Case Report Dent.

[3] Sineedi FA, Aruveetil YA, Kavarodi AM, Harbi SO ( 2018). Bilocular unicystic ameloblastoma of the mandible in a 9 yr old child – a diagnostic and management dilemma. Saudi Dent J.

[4] Andrade NN, Shetye SP, Mhatre TS ( 2013). Trends in Pediatric Ameloblastoma and its Management: A 15year Indian Experience. J Maxillofac Oral Surg.

[5] Nagarajappa AK, Pandya D, Jain N ( 2015). An Unusual case of ameloblastoma arising from dentigerous cyst. J Orofac Res.

[6] Reichart PA, Philipsen HP (2004). Odontogenic tumors and allied lesions.

[7] Marx RE, Stern D (2012). Odontogenic tumors: hamartomas and neoplasms. In oral and maxillofacial pathology: a rationale for diagnosis and treatment.

[8] Dolanmaz D, Etoz OA, Pampu A, Kalayci A, Gunhan O ( 2011). Marsupialization of unicystic ameloblastoma: A conservative approach for aggressive odontogenic tumors. Indian J Dent Res.

[9] Kumar KR, George GB, Padiyath S, Rupak S ( 2012). Mural unicystic ameloblastoma crossing the midline: a rare case report. Int J Odontostomat.

[10] Marimuthu L, Kumar S, Shenoy V, Afradh M, Paranthaman A ( 2020). Mural variant of unicysticameloblastoma in a pediatricpatient: arare case report. Cureus.

[11] Kim SW, Jee YJ, Lee DW, Kim HK ( 2018). Conservative surgical treatment for ameloblastoma: a report of three cases. J Korean Assoc Oral Maxillofac Surg.

